# Supplementary material for: Locally Adapted and Organically Grown Landrace and Ancient Spring Cereals—A Unique Source of Minerals in the Human Diet
Source: Foods. 2021 Feb 11;10(2):393. doi: 10.3390/foods10020393 (PMC7916914; doi:10.3390/foods10020393)
Supplement: Supplementary file 1 [file foods-10-00393-s001.pdf]

Table S1. Mean values over three years of content of the various minerals, yield and grain protein content for the genotypes grown at each location. Genotypes grown in all four localities are marked with bold text.

Source	Zn (10[1])	S (10[3])	P (10[3])	Na (10[1])	Mn (10[1])	Mg (10[3])	K (10[3])	Fe (10[1])	Cu	Ca (10[2])	Yield (kg ha^{-1})	Protein (%)
Ekhaga												
Algot	4.10	1.56	4.26	3.15	3.30	1.32	3.82	4.27	8.17	6.01	3190	12.4
Balder	3.67	1.45	3.95	3.00	1.10	1.17	4.00	3.55	5.19	3.05	3200	11.3
Bambu	4.55	2.16	5.70	1.50	5.68	1.54	3.85	6.49	6.02	6.58	2630	12.8
Dacke	4.10	1.55	4.21	2.00	3.62	1.31	3.60	3.82	5.26	3.96	3370	13.5
**Diamant brun**	**5.65**	**1.71**	**4.29**	**2.53**	**4.17**	**1.31**	**3.43**	**5.35**	**6.62**	**5.35**	**2780**	**13.6**
Domen	4.12	1.52	4.02	3.00	0.97	1.14	3.97	4.53	7.16	3.99	2680	11.5
**Ella**	**4.73**	**1.55**	**4.10**	**3.27**	**3.58**	**1.30**	**3.59**	**4.64**	**6.84**	**5.00**	**3440**	**12.0**
**Emmer Gotland**	**5.62**	**1.82**	**4.86**	**1.77**	**3.80**	**1.49**	**4.00**	**4.82**	**7.28**	**3.71**	**2500**	**13.2**
**Engelbrekt**	**3.98**	**2.01**	**5.49**	**4.45**	**4.72**	**1.47**	**3.62**	**5.24**	**6.13**	**5.97**	**3460**	**12.2**
**Hulless 6row barley**	**4.67**	**1.56**	**4.38**	**5.42**	**1.73**	**1.33**	**4.21**	**5.18**	**5.53**	**5.58**	**2220**	**12.5**
**Hulless 2row barley**	**4.70**	**1.71**	**5.14**	**5.55**	**1.72**	**1.42**	**4.63**	**5.08**	**6.61**	**5.80**	**2520**	**14.7**
**Hulless oats**	**4.10**	**2.01**	**5.07**	**1.75**	**5.13**	**1.45**	**3.40**	**5.21**	**5.69**	**7.90**	**2280**	**15.2**
**Ingrid**	**3.42**	**1.47**	**3.66**	**2.95**	**0.95**	**1.13**	**3.56**	**2.98**	**5.59**	**3.18**	**3160**	**11.0**
**Jusso**	**4.95**	**1.60**	**4.03**	**2.30**	**2.90**	**1.08**	**4.26**	**5.35**	**6.24**	**4.82**	**1810**	**11.9**
Kajsa	4.40	1.51	4.37	3.62	1.20	1.18	3.73	4.09	6.14	4.06	2430	12.8
Kärn	4.00	1.52	4.30	4.38	3.70	1.24	3.82	4.19	5.78	4.86	3690	12.0
Landrace Dalarna	3.80	1.50	4.19	2.23	3.53	1.21	4.12	3.70	5.37	4.33	3750	12.6
Landrace Halland	3.78	1.52	4.51	2.43	3.42	1.27	4.15	3.93	5.29	4.93	3680	12.3
Orion	4.68	2.27	5.94	1.87	5.05	1.61	4.01	6.38	6.90	6.59	2060	12.7
Prins	4.18	1.58	3.95	2.80	3.05	1.26	3.71	4.69	5.70	3.97	3240	12.6
Sisu	4.05	2.03	5.21	1.73	4.30	1.49	4.02	5.06	5.62	6.71	3420	11.5
Sol	3.98	2.17	5.38	1.65	4.65	1.38	4.12	5.22	5.78	6.68	3450	11.9
**Spelt wheat Gotland**	**4.83**	**1.81**	**4.97**	**1.85**	**3.93**	**1.50**	**3.71**	**4.75**	**7.60**	**2.92**	**3150**	**14.9**
**Virma**	**4.18**	**2.21**	**5.38**	**2.63**	**5.25**	**1.50**	**4.06**	**5.73**	**6.46**	**6.76**	**3640**	**12.2**
**Öland**	**5.20**	**1.76**	**5.06**	**2.27**	**3.77**	**1.49**	**3.74**	**5.71**	**6.50**	**5.37**	**3090**	**13.2**
Krusenberg												
Algot	4.10	1.52	396	0.97	4.07	1.19	3.82	4.35	5.41	5.50	2620	11.7
Aurore	3.47	1.42	3.91	1.27	3.92	1.15	3.87	3.83	4.77	4.89	2250	12.1
Balder	2.92	1.33	3.60	2.80	1.32	1.07	3.42	3.33	4.01	2.92	2870	10.5
Dacke	3.95	1.50	3.83	1.30	3.73	1.17	3.83	3.87	4.87	4.75	2270	11.8
**Diamant brun**	**4.05**	**1.43**	**4.09**	**1.43**	**4.68**	**1.12**	**3.81**	**4.59**	**5.18**	**5.66**	**2140**	**11.3**
**Ella**	**3.62**	**1.45**	**4.05**	**1.55**	**4.40**	**1.20**	**3.81**	**4.34**	**4.79**	**4.86**	**2470**	**11.1**
**Emmer Gotland**	**4.77**	**1.60**	**4.66**	**1.95**	**4.57**	**1.36**	**4.18**	**4.39**	**5.92**	**3.51**	**1880**	**12.3**
**Engelbrekt**	**4.37**	**1.93**	**5.29**	**2.28**	**5.25**	**1.48**	**3.63**	**5.40**	**5.41**	**5.53**	**2050**	**11.8**
Extra klock	4.32	2–03	5.56	1.38	5.43	1.54	4.06	5.15	4.75	5.36	1860	11.2
**Hulless 6row barley**	**3.62**	**1.52**	**4.29**	**3.18**	**2.32**	**1.26**	**4.05**	**3.55**	**4.62**	**4.70**	**2510**	**11.5**
**Hulless 2row barley**	**3.23**	**1.59**	**4.59**	**3.95**	**2.48**	**1.29**	**4.04**	**3.52**	**4.72**	**4.30**	**2630**	**13.2**
**Hulless oats**	**4.15**	**2.12**	**5.40**	**1.70**	**6.00**	**1.60**	**3.98**	**3.93**	**4.00**	**9.20**	**1080**	**12.5**
**Ingrid**	**2.30**	**1.36**	**3.35**	**2.05**	**0.92**	**1.04**	**3.17**	**2.71**	**3.66**	**2.99**	**3350**	**10.8**
**Jusso**	**4.03**	**1.37**	**3.98**	**1.00**	**3.90**	**1.15**	**4.21**	**4.11**	**4.72**	**4.02**	**2410**	**10.7**
Kajsa	3.17	1.34	4.18	2.88	1.65	1.14	3.46	3.41	5.02	3.35	2670	11.9
Kärn	3.57	1.43	3.95	1.27	4.20	1.16	3.83	3.98	4.33	4.94	2760	11.1
Landrace Dalarna	3.73	1.36	3.89	1.72	4.75	1.14	3.95	3.89	4.41	4.45	2570	11.1
Landrace Halland	3.75	1.43	3.94	2.32	4.80	1.15	3.96	4.28	5.04	4.56	2390	11.1
Orion	4.77	2.13	5.78	1.33	5.67	1.63	3.92	5.39	5.81	5.96	1240	12.9
Seger	4.97	2.24	5.69	1.12	5.93	1.65	4.16	5.30	5.61	6.07	2020	11.1
Sol	4.35	2.08	5.43	1.23	5.45	1.57	4.18	5.01	5.31	5.95	2150	10.5

**Spelt wheat Gotland**	**4.43**	**1.83**	**4.89**	**1.28**	**5.05**	**1.48**	**3.84**	**4.84**	**5.48**	**3.94**	**2250**	**12.6**
Svanhals	2.88	1.44	4.02	2.27	1.32	1.22	3.17	3.19	4.35	3.12	2680	12.5
**Virma**	**4.37**	**1.86**	**4.93**	**1.07**	**6.05**	**1.39**	**3.84**	**4.63**	**4.80**	**5.39**	**1540**	**10.8**
**Öland**	**3.97**	**1.50**	**4.25**	**1.02**	**4.70**	**1.22**	**3.71**	**4.76**	**4.74**	**5.27**	**2690**	**12.3**
Gotland												
Argus	3.55	2.10	5.91	1.85	2.73	1.55	3.97	4.27	4.73	6.62	2820	10.2
Atle	4.10	1.60	4.41	4.48	1.25	1.38	4.04	3.73	5.12	5.85	2390	13.4
Blenda	3.67	2.13	5.45	2.07	1.97	1.48	3.86	4.36	4.36	7.30	3980	11.5
**Diamant brun**	**4.87**	**1.66**	**4.57**	**2.00**	**1.38**	**1.38**	**3.44**	**3.93**	**4.93**	**5.87**	**2650**	**13.3**
Dragon	4.00	1.72	4.67	1.85	1.37	1.48	4.07	3.20	6.03	4.30	3340	13.3
**Ella**	**4.47**	**1.57**	**4.52**	**1.87**	**0.95**	**1.40**	**3.59**	**3.81**	**5.33**	**5.25**	**3010**	**13.1**
**Emmer Gotland**	**5.15**	**1.57**	**5.76**	**2.55**	**1.57**	**1.68**	**4.47**	**4.15**	**6.04**	**4.41**	**3270**	**14.0**
**Engelbrekt**	**3.77**	**2.03**	**5.56**	**1.48**	**1.68**	**1.52**	**3.73**	**4.45**	**5.13**	**6.73**	**3650**	**11.0**
Gotlandskorn	2.95	1.25	3.78	3.62	0.52	1.06	3.41	3.67	4.37	3.46	4560	10.9
Gullkorn	3.70	1.46	4.48	4.07	0.52	1.20	3.66	3.79	5.29	3.97	2960	13.5
**Hulless 6row barley**	**3.78**	**1.54**	**4.77**	**8.37**	**0.85**	**1.30**	**4.32**	**4.20**	**4.80**	**5.78**	**2850**	**12.8**
**Hulless 2row barley**	**3.98**	**1.67**	**5.11**	**7.93**	**1.22**	**1.37**	**4.18**	**4.81**	**5.62**	**6.06**	**3160**	**15.0**
**Hulless oats**	**3.33**	**1.85**	**5.12**	**1.88**	**2.60**	**1.44**	**3.85**	**4.02**	**4.56**	**7.55**	**2630**	**13.8**
**Ingrid**	**2.63**	**1.31**	**4.10**	**3.90**	**0.60**	**1.10**	**3.63**	**3.88**	**4.54**	**3.24**	**4650**	**11.1**
**Jusso**	**4.52**	**1.70**	**4.68**	**2.77**	**1.45**	**1.28**	**4.62**	**4.30**	**5.82**	**5.57**	**1890**	**12.9**
Landrace Dalarna	4.75	1.73	4.59	3.22	1.47	1.43	3.79	4.31	5.70	6.26	2710	13.8
Orion	4.25	2.21	5.57	2.23	2.25	1.53	3.88	5.41	5.59	7.15	2030	12.1
Prins	4.05	1.62	4.38	2.20	0.98	1.41	3.83	3.79	5.00	4.71	3170	14.0
Rika	3.38	1.31	3.99	4.23	0.42	1.11	3.54	3.90	5.53	3.31	4360	11.3
**Spelt wheat Gotland**	**4.68**	**1.91**	**5.37**	**2.45**	**1.78**	**1.60**	**3.94**	**4.03**	**6.85**	**3.27**	**3090**	**14.0**
Spelt wheat Gotland d	5.02	1.97	5.20	2.43	1.45	1.59	3.79	3.49	7.36	3.81	3410	13.7
Summer oats	3.83	2.22	6.00	2.58	1.80	1.64	3.71	5.14	6.18	7.45	3370	7.8
Ur gotland	4.02	2.36	6.03	3.83	1.60	1.73	4.12	5.21	6.02	7.97	2210	10.6
**Virma**	**3.82**	**2.13**	**5.26**	**1.83**	**1.92**	**1.46**	**3.90**	**4.22**	**4.91**	**7.40**	**3330**	**11.7**
**Öland**	**4.13**	**1.66**	**4.57**	**1.35**	**1.40**	**1.43**	**3.57**	**4.44**	**4.95**	**4.77**	**2560**	**14.7**
Alnarp												
Algot	3.05	1.28	4.11	2.72	2.33	1.24	4.03	3.33	4.04	4.58	3740	11.0
Alva	2.58	1.23	4.25	4.82	0.92	1.14	4.04	3.90	4.06	3.72	4060	9.3
Atle	3.02	1.33	4.09	2.07	2.27	1.22	4.04	3.28	3.94	4.82	3500	10.8
Atson	2.88	1.44	4.41	1.80	1.83	1.31	4.10	3.95	4.57	4.23	3560	11.1
**Diamant brun**	**3.82**	**1.49**	**4.48**	**1.68**	**3.42**	**1.32**	**3.70**	**4.72**	**4.53**	**5.41**	**2790**	**11.6**
Dragon	2.60	1.26	4.05	2.03	2.20	1.23	4.08	3.29	3.76	4.44	4130	10.6
**Ella**	**3.23**	**1.41**	**4.41**	**1.75**	**3.10**	**1.30**	**3.79**	**3.54**	**4.85**	**4.71**	**3670**	**11.4**
**Emmer Gotland**	**3.75**	**1.44**	**4.70**	**28.5**	**2.43**	**1.30**	**3.93**	**4.16**	**5.28**	**4.41**	**2850**	**11.9**
**Engelbrekt**	**3.68**	**1.81**	**5.29**	**2.68**	**3.08**	**1.38**	**3.67**	**4.35**	**3.71**	**6.37**	**3710**	**10.6**
Gotlandskorn	2.35	1.15	4.05	4.30	2.18	1.09	4.09	3.50	3.95	3.83	4470	9.5
**Hulless 6row barley**	**3.08**	**1.51**	**4.84**	**5.78**	**1.32**	**1.31**	**4.29**	**4.36**	**4.49**	**5.87**	**2370**	**11.8**
**Hulless 2row barley**	**3.07**	**1.45**	**5.13**	**5.73**	**1.42**	**1.35**	**4.40**	**4.67**	**5.09**	**5.79**	**2380**	**13.1**
**Hulless oats**	**2.65**	**1.53**	**4.45**	**3.08**	**2.93**	**1.20**	**3.59**	**3.86**	**3.46**	**7.72**	**2850**	**10.8**
**Ingrid**	**2.42**	**1.19**	**3.82**	**4.37**	**0.65**	**1.04**	**3.77**	**3.03**	**4.05**	**3.47**	**4540**	**9.4**
**Jusso**	**3.17**	**1.32**	**4.18**	**1.93**	**1.33**	**1.09**	**4.64**	**3.54**	**4.73**	**4.34**	**3210**	**10.0**
Klock	3.58	1.88	5.29	2.57	3.37	1.40	3.62	4.04	3.94	6.13	2920	11.6
Lina	2.43	1.13	4.12	4.32	0.82	1.09	4.14	3.25	4.06	4.92	4090	9.5
Osmo	34.8	2.00	5.47	2.47	3.40	1.46	3.66	4.91	3.97	6.88	2830	13.3
Palu	3.85	1.83	5.11	2.28	2.68	1.36	3.87	3.94	3.95	6.91	3600	10.7
Seger	3.85	1.81	5.26	2.75	3.58	1.46	3.66	4.91	3.74	6.22	3160	10.7
Selma	3.40	1.64	4.81	3.42	2.72	1.29	3.55	4.18	3.45	7.23	3890	10.5
**Spelt wheat Gotland**	**3.60**	**1.58**	**4.96**	**2.78**	**2.45**	**1.39**	**3.78**	**4.47**	**5.80**	**3.00**	**3080**	**12.7**
Walter	2.75	1.37	4.07	1.93	1.77	1.23	3.79	3.78	3.85	4.03	3740	11.4
**Virma**	**3.23**	**1.80**	**4.90**	**2.00**	**3.12**	**1.31**	**3.75**	**3.61**	**3.71**	**6.94**	**3820**	**10.4**
**Öland**	**3.32**	**1.52**	**4.81**	**1.45**	**2.20**	**1.46**	**3.75**	**4.61**	**4.78**	**5.20**	**3460**	**12.0**

Table S2. Pearson correlation coefficients between evaluated minerals.

Source	Zn	S	P	Na	Mn	Mg	K	Fe	Cu
S	0.51 ***								
P	0.34 ***	0.69 ***							
Na	0.09	−0.11	0.01						
Mn	0.25 ***	0.33 ***	0.26 ***	−0.40 ***					
Mg	0.49 ***	0.71 ***	0.89 ***	−0.02	0.25 ***				
K	0.09	−0.03	0.22 ***	0.13 *	0.10	0.14 *			
Fe	0.52 ***	0.60 ***	0.43 ***	0.03	0.37 ***	0.45 ***	−0.03		
Cu	0.63 ***	0.44 ***	0.19 ***	0.15 *	0.02	0.34 ***	−0.17 ***	0.54 ***	
Ca	0.30 ***	0.59 ***	0.55 ***	0.15 **	0.25 ***	0.50 ***	0.23 ***	0.36 ***	0.07

*,**, *** = Significant at $p < 0.05$, 0.01 and 0.005.

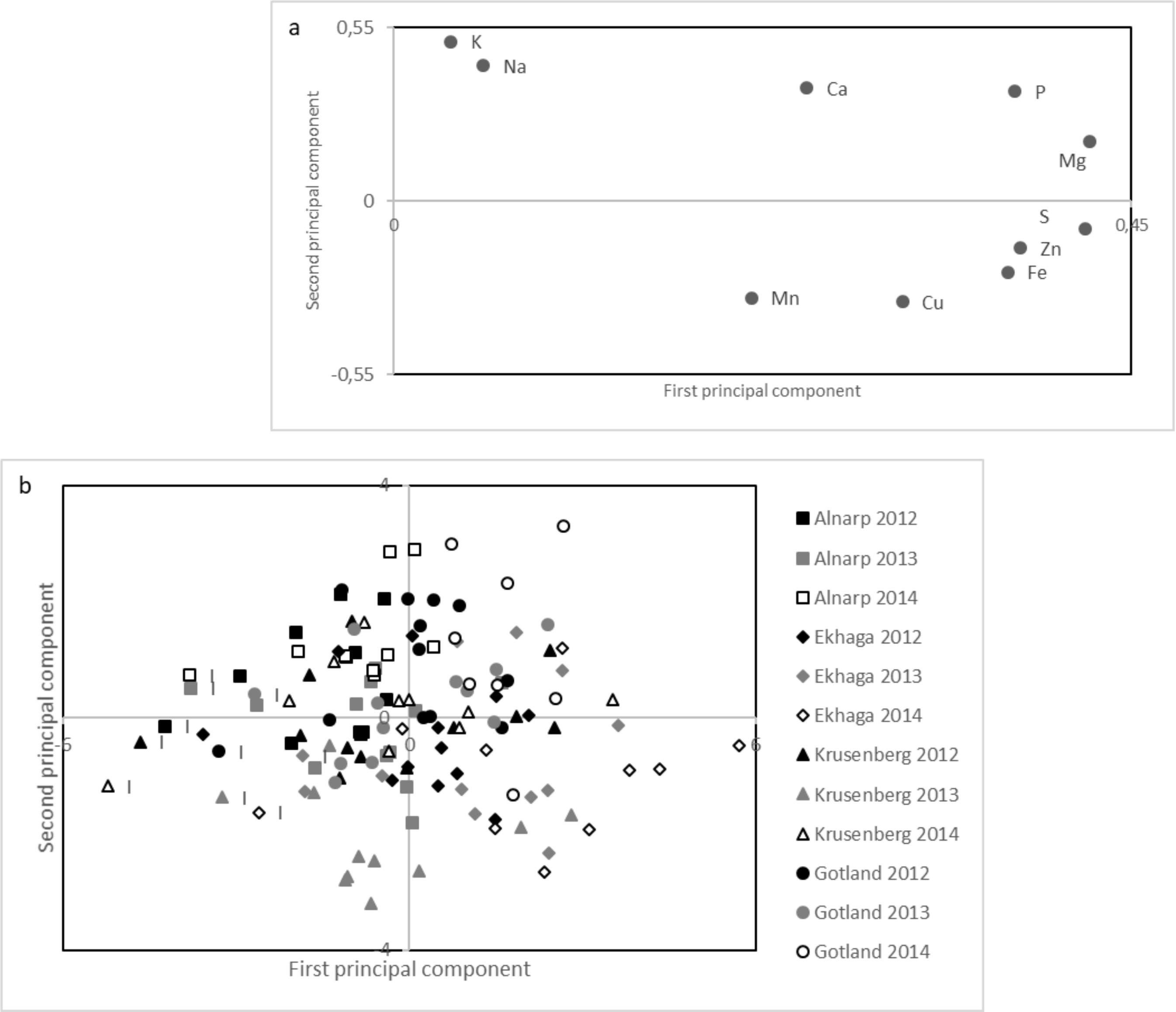

Figure S1. Score (**a**) and loading (**b**) plot from principal component analysis of mineral content in organically grown and locally adapted spring cereals grown at different localities during different years. I indicates the spring barley cultivar Ingrid. First principal component explained 35.0% of the variation and the second principal component explained 17.2% of the variation.
